# Supplementary material for: The Relationship of Temperament and Character, Parental Stress, and Mental Health Problems with Attachment Disorders among Children
Source: Int J Environ Res Public Health. 2022 Nov 22;19(23):15458. doi: 10.3390/ijerph192315458 (PMC9737910; doi:10.3390/ijerph192315458)
Supplement: Supplementary file 1 [file ijerph-19-15458-s001.zip › ijerph-1997932-supplementary.pdf]

Table S1. Attrition bias analysis of included and excluded participants (N = 156).

Variable	Excluded (n = 31)	Included (n = 125)	Test statistic
Age in years (M [SD])	7.00 (1.46)	7.14 (1.38)	$t(31.42) = -0.55, p = 0.59$
Gender (n [%])			χ^2 (1) = 4.36, p = 0.02*
Female	4 (12.9)	49 (39.2)	
Male	22 (71.0)	76 (60.8)	
Nationality (Swiss)	11 (45.9)	66 (52.8)	$\chi^2(1) = 0.34, p = 0.56$
Mental health problems (M [SD])			
Internalizing problems	58.56 (9.90)	58.96 (11.35)	$t(9.58) = -0.12, p = 0.91$
Externalizing problems	64.11 (11.73)	61.68 (11.27)	$t(9.10) = 0.60, p = 0.56$
Total problem behavior	63.33 (12.88)	62.21 (11.39)	$t(8.92) = 0.25, p = 0.80$
Personality traits (M [SD])			
Novelty Seeking	63.17 (10.44)	52.82 (12.41)	$t(5.70) = 2.35, p = 0.06$
Harm Avoidance	49.00 (9.03)	52.05 (10.77)	$t(5.70) = -0.80, p = 0.46$
Reward Dependence	50.67 (17.51)	44.82 (11.91)	$t(5.22) = 0.81, p = 0.45$
Persistence	38.00 (15.05)	44.32 (11.54)	$t(5.29) = -1.01, p = 0.35$
Self-directedness	39.83 (6.31)	43.74 (11.64)	$t(6.76) = -1.41, p = 0.20$
Cooperativeness	43.83 (20.12)	44.56 (12.89)	$t(5.20) = -0.09, p = 0.93$
Self-transcendence	41.50 (11.02)	45.71 (10.06)	$t(5.41) = -0.92, p = 0.40$
Relationship problems (M [SD])			
Total scale	6.44 (5.59)	4.65 (4.91)	$t(8.91) = 0.94, p = 0.37$
Disinhibited subscale	4.11 (3.89)	2.35 (2.83)	$t(8.62) = 1.33, p = 0.22$
Inhibited subscale	2.33 (2.45)	2.30 (2.98)	$t(9.79) = 0.04, p = 0.97$
Attachment disorder (n [%])	2 (6.5)	33 (26.4)	$\chi^2(1) = 0.00, p = .65$
Attachment disorder type (n [%])			$\chi^2(3) = 1.78, p = .36$
None	7 (22.6)	92 (73.6)	
Inhibited	1 (3.2)	9 (7.2)	
Disinhibited	1 (3.2)	11 (8.8)	
Mixed-type	0 (0)	13 (10.4)	
Attachment relationship type			$\chi^2(5) = 6.19, p = .65$
Secure	4 (12.9)	37 (29.6)	
Ambivalent	4 (12.9)	19 (15.2)	
Avoidant	1 (3.2)	22 (17.6)	
Disorganized	1 (3.2)	27 (27.0)	
Parental stress (M [SD])	38.33 (11.52)	38.56 (9.21)	$t(5.31) = -0.05, p = 0.96$

Note. The number of participants does not add up to n = 28 for excluded participants in the categorical variables due to missing data. *$p < 0.05$.
